# Optimizing the Caloric Properties of Cu-Doped Ni–Mn–Ga Alloys

**DOI:** 10.3390/ma13020419

**Published:** 2020-01-16

**Authors:** Concepcio Seguí, Joan Torrens-Serra, Eduard Cesari, Patricia Lázpita

**Affiliations:** 1 Departament de Física, Universitat de les Illes Balears, E07122 Palma de Mallorca, Spain; j.torrens@uib.es (J.T.-S.); eduard.cesari@uib.cat (E.C.); 2 Departamento de Electricidad y Electrónica, Facultad de Ciencia y Tecnología, UPV/EHU, E48940 Leioa, Spain; patricia.lazpita@ehu.eus

**Keywords:** ferromagnetic shape memory alloys, Cu-doped Ni–Mn–Ga, entropy, hysteresis, magnetocaloric effect, elastocaloric effect

## Abstract

With the purpose to optimize the functional properties of Heusler alloys for their use in solid-state refrigeration, the characteristics of the martensitic and magnetic transitions undergone by Ni_50_Mn_25−*x*_Ga_25_Cu*_x_* (*x* = 3–11) alloys have been studied. The results reveal that, for a Cu content of *x* = 5.5–7.5, a magnetostructural transition between paramagnetic austenite and ferromagnetic martensite takes place. In such a case, magnetic field and stress act in the same sense, lowering the critical combined fields to induce the transformation; moreover, magnetocaloric and elastocaloric effects are both direct, suggesting the use of combined fields to improve the overall refrigeration capacity of the alloy. Within this range of compositions, the measured transformation entropy is increased owing to the magnetic contribution to entropy, showing a maximum at composition *x* = 6, in which the magnetization jump at the transformation is the largest of the set. At the same time, the temperature hysteresis of the transformation displays a minimum at *x* = 6, attributed to the optimal lattice compatibility between austenite and martensite. We show that, among this system, the optimal caloric performance is found for the *x* = 6 composition, which displays high isothermal entropy changes (−36 J·kg^−1^·K^−1^ under 5 T and −8.5 J·kg^−1^·K^−1^ under 50 MPa), suitable working temperature (300 K), and low thermal hysteresis (3 K).

## 1. Introduction

Heusler-type ferromagnetic shape memory alloys (FSMAs) undergo a structural, martensitic transformation (MT), where the high and low temperature phases do often present different magnetic orders [1,2]. The coupling of the structural and magnetic transitions gives place to a number of interesting multifunctional properties such as the magnetic shape memory effect and magnetic superelasticity, magnetocaloric effect, magnetoresistance, and so on [3]. These effects are even more promising for practical applications than the magnetic field induced strains by reorientation of martensitic variants, which, to date, have attracted the greatest interest exploitation. To the extent that those effects rely on the induction of the phase transformation by an applied magnetic field (*H*), the change in the transformation temperature is obtained using the Claussius–Clapeyron equation:

(1) $$\frac{dT}{dH}=-\mu_{o}\frac{\Delta M}{\Delta S}$$

where *µ*_o_ is the vacuum magnetic permeability, Δ*S* is the entropy change, and Δ*M* is the magnetization change associated to the transformation. It follows from this equation that only FSMAs for which the structural transformation is accompanied by significant magnetization change are candidates for the related applications. That is, the case of the as-called *metamagnetic* shape memory alloys (MMSMAs), such as Ni–Mn–X (X = In, Sn, Sb) [1,2,3], which undergo martensitic transformation from ferromagnetic austenite (*A_ferro_*) to low magnetization martensite (*m_weak_*), accompanied by large magnetization drop. Instead, for ternary Ni–Mn–Ga alloys, austenite and martensite usually have similar magnetic order (thus, Δ*M* is small) [4]. In recent years, it has been proven that Co doping increases the ferromagnetic coupling of the austenite and weakens the magnetic interactions of the martensite, so that, for a limited range of Co content, Ni_50−*x*_Co*_x_*Mn_30_Ga_20_ (*x* = 6–9), Ni–Mn–Ga–Co alloys show the aforementioned metamagnetic behavior with significant negative Δ*M* [5,6].

It is crucial to keep in mind that, according to Equation (1), for a metamagnetic *A_ferro_*⟶*m_weak_* MT with Δ*M* < 0, being Δ*S* < 0, it follows that (d*T*/d*H*) < 0, thus the applied magnetic field decreases the transformation temperatures favoring reverse MT [1,2,3]. Conversely, in the case of paramagnetic austenite to ferromagnetic martensite transition (*A_para_*⟶*m_ferro_*), forward MT is accompanied by positive Δ*M*, so that (d*T*/d*H*) > 0 and forward MT is stimulated when magnetic field is applied. It is interesting to note that, only in the latter case, the magnetic field and stress act in the same sense, thus cooperating to induce the MT under lower values of one or another applied field.

Simultaneous action of magnetic and mechanical fields could not only improve the actuation performance of the alloys, but also result in better prospects of using these alloys for solid state refrigeration, as magnetostructural transformations are accompanied by several caloric effects [7]. Magnetocaloric effect (MCE) [7,8,9] is characterized by positive isothermal entropy change induced by the magnetic field (Δ*S_M_*) in the case of the ferromagnetic to paramagnetic (or weak magnetic) transition, as undergone by common MMSMAs (inverse MCE) [9,10]; on the contrary, paramagnetic to ferromagnetic magnetostructural transformation is accompanied by direct (or conventional) MCE, which yields negative Δ*S_M_* upon increasing the magnetic field. On the other hand, the elastocaloric effect (ECE) is a common feature of shape memory alloys (SMAs), arising from the reversible stress-induced MT, and produces the negative isothermal entropy change with applied stress (Δ*S_E_*) [10,11,12]. That is, while ECE is always direct, regarding FSMAs, MCE is only direct for materials undergoing the positive magnetization jump during the magnetostructural transition; therefore, only in that case, the global caloric response can be improved by combining MCE and ECE [13].

Besides Δ*M*, the other component in Equation (1) is the transformation entropy change (Δ*S*). It is widely accepted that Δ*S* can be considered as the addition of lattice (or structural) and magnetic terms (the small electronic contribution is usually neglegted) [14]:

(2) $$\Delta S^{A↔m}=\Delta S_{str}^{A↔m}+\Delta S_{mag}^{A↔m},$$

where $\Delta S_{str}^{A\to m}<0$ and $\Delta S_{str}^{m\to A}=\left|\Delta S_{str}^{A\to m}\right|>0$, while the sign and value of $\Delta S_{mag}^{A\to m}=-\Delta S_{mag}^{m\to A}$ depend on the magnetic exchange interaction between the related phases. It can be expected that $\Delta S_{mag}^{A↔m}=0$ if neither austenite nor martensite exhibit magnetic order; $\Delta S_{mag}^{A\to m}>0$ (and $\Delta S_{mag}^{m\to A}<0$) if the magnetization decreases upon MT, as for *A_ferro_*↔*m_weak_*. Conversely, $\Delta S_{mag}^{A\to m}<0$ (and $\Delta S_{mag}^{m\to A}>0$) for *A_para_*↔*m_ferro_*, while $\Delta S_{mag}^{A\to m}$ could be either positive or negative depending on the relative value of magnetization of austenite and martensite in the case of *A_ferro_*↔*m_ferro_* MT.

For the sake of simplicity, we will use a unique expression referring to the absolute values of the entropy contributions:

(3) $$\left|\Delta S\right|=\left|\Delta S_{str}\right|\pm \left|\Delta S_{mag}\right|,$$

where addition corresponds to *A_para_*↔*m_ferro_* and subtraction to *A_ferro_*↔*m_weak_* transitions. That means that the (absolute) value of Δ*S* depends on the type of magnetic exchange; as a general trend, without underestimating the variations of Δ*S_str_* due to composition and/or applied fields, |ΔS| will be higher for MTs with positive ΔM than for those with negative Δ*M*. While, according to the Claussius–Clapeyron equations, a large |ΔS| implies that the fields required to induce the MT would be large. It is also true that the transformation entropy change is the bound value that isothermal entropy associated to caloric effects (Δ*S_M_*, Δ*S_E_*) can reach; hence, the larger the |ΔS|, the greater would be the refrigeration capacity of the alloys [11].

Table 1 summarizes the above described characteristics for *A_ferro_*→*m_weak_* MT (hereafter, conventional metamagnetism) and *A_para_*→*m_ferro_* MT (hereafter, direct metamagnetism).

In addition to the maximum value of entropy, other factors associated with magnetostructural transformations determine the effective applicability of a material in a refrigeration device. Among them, hysteresis, inherent to first-order phase transformations, has to be included. Particularly, in most common caloric refrigeration prototypes, it is seen as a drawback, and thus its reduction is a major goal in the search for solid-state refrigerant materials [15,16].

To date, not many FSMAs undergoing direct metamagnetism are known. It has been previously reported that Cu-doping of Ni_2_MnGa alloys allows smooth adjustment of the magnetic and structural transition temperatures, making it possible to find different structures and magnetic behaviors across the MT in both Ni_2_MnGa_1−*y*_Cu*_y_* and Ni_2_Mn_1−*x*_Cu*_x_*Ga systems [17,18,19,20]. Cu substituting Mn is found to increase the MT temperatures, but Cu weakens the magnetic interactions, leading to a decrease of Curie temperature [18,20]. As a result, the structural and magnetic transitions can be tuned in such a way that *A_para_*⟶*m_ferro_* transition occurs for selected composition ranges of the Ni_2_Mn_1−*x*_Cu*_x_*Ga.

In this work, the characteristics of the structural and magnetic transitions undergone by Ni_50_Mn_25−*x*_Ga_25_Cu*_x_* (*x* = 3–11) alloys were studied. The composition dependence of the transition temperatures, transformation entropy change, and temperature hysteresis were analyzed. From the results, the compositions for which the above alloys show direct metamagnetic behavior are determined, and the transformation entropy change is correlated with the magnetic contribution. The transformation hysteresis results are also composition dependent and, in analyzing this behavior, the martensite structures are also taken into account. For selected compositions within the metamagnetic window, the behavior of the magnetostructural transformation under magnetic field was also studied. To assess the applicability of these alloys, the magnetocaloric and elastocaloric effects were evaluated, and the results are discussed in relation to the characteristics of the magnetostructural transformations.

## 2. Experimental Details

Polycrystalline Ni_50_Mn_25−*x*_Ga_25_Cu*_x_* (*x* = 3–11) alloy ingots were prepared by induction melting in argon atmosphere, using high purity elemental metals. The ingots were melted several times and homogenized for 24 h at 1170 K in a vacuum quartz tube, followed by quench in water at room temperature.

The MT characteristics, transformation temperatures, and exchanged heat were obtained from differential scanning calorimetry (DSC) measurements at a heating rate of 1 K/min (DSC 2920, TA Instruments, New Castle, DE, USA), while the magnetic transition temperatures were obtained from thermogravimetric analysis (SDT Q600, TA Instruments, New Castle, DE, USA) performed under the influence of a permanent magnet, which exerts a magnetic field of about 100 mT. As the measured weight increases when the samples are magnetized, the apparent weight changes correspond to the evolution of magnetization as a function of temperature [5,6]. For the selected compositions, magnetization versus temperature curves, M(T), were recorded at 3 K/min under magnetic fields up to 7 T using a vibrating sample magnetometer (VSM; magnetic platform from Cryogenic Ltd. CFMS, London, UK); similarly, strain versus temperature curves, ε(T), under different loads up to 50 MPa were measured using a dynamic mechanical analyzer (DMA Q800, TA Instruments, New Castle, DE, USA) in three-point-bending configuration at 5 K/min. The adiabatic temperature changes associated with the magnetocaloric effect was measured for applied magnetics fields of 1 and 2 T at different temperatures in an own-built set up specially designed for this purpose (see the work of [21] for details). The adiabatic entropy associated with the elastocaloric effect was obtained during compressive loading and unloading up to 100 MPa at a strain rate $\dot{\epsilon }=0.01\;s^{-1}$. The measurement was performed using a thermocouple attached to a rectangular cuboid-shaped sample at a temperature of T = 310 K.

Structural determination of the phases was carried out using powder X-ray diffraction (XRD BRUKER D8 Advance, Billerica, MA, USA). Diffraction patterns were taken at different temperatures, covering the whole transformation range (310–240 K).

## 3. Results and Discussion

### 3.1. Structural and Magnetic Transitions

**Figure 1:**
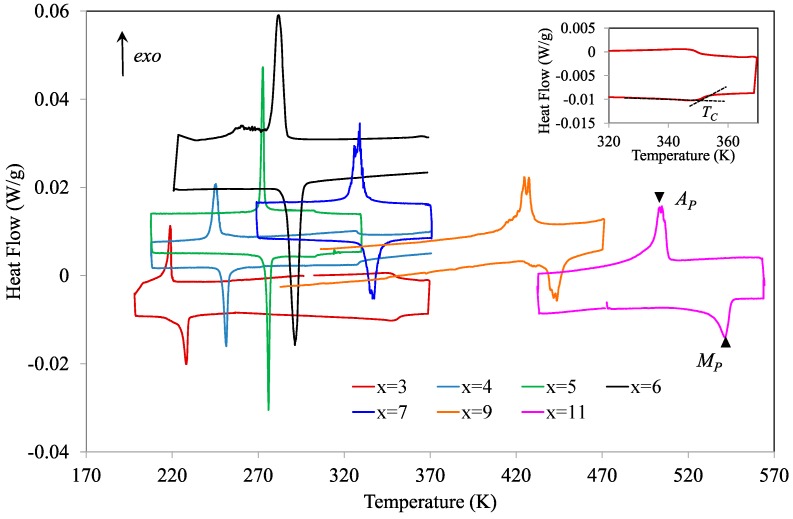
Differential scanning calorimetry (DSC) curves obtained during cooling/heating cycles for the complete set of studied alloys. The characteristic temperatures, *M_P_* for the forward and *A_p_* for the reverse martensitic transformations (MTs), are also indicated. The inset shows the determination of the Curie temperature of austenite, *T_C_^A^*, for the alloy with *x* = 3.

Figure 1 shows the DSC curves obtained for the complete set of alloys during cooling/heating cycles performed at a rate of 1 K/min. The curves exhibit peaks corresponding to the austenite↔martensite transformations and the temperatures for which the heat flow is maximum/minimum are taken as characteristics of the structural transition, namely *M_P_* for the forward and *A_p_* for the reverse MTs.

**Figure 2:**
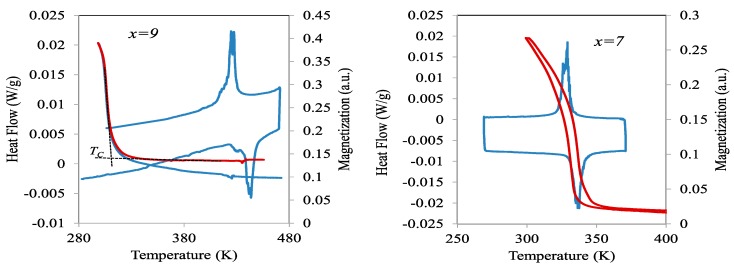
Thermogravimetric curves obtained during cooling/heating cycles performed under a permanent magnet for alloys with *x* = 9 and *x* = 7. The corresponding DSC curves are also shown.

For alloys with Cu content less than 6, DSC curves show, in the austenite temperature range, an inflection that can be clearly attributed to the ferromagnetic transition of the parent phase; an example is shown in the inset of Figure 1 for *x* = 3, where the determination of the Curie temperature of austenite, *T_C_^A^*, is also indicated. For the other alloys, such an inflection is not observed, but the magnetic transition can be evidenced by thermogravimetric measurements under a permanent magnet. Examples are shown in Figure 2 for alloys with *x* = 7 and *x* = 9. However, while for *x* = 9, a non-hysteretic magnetization increase is observed in the martensitic region—thus corresponding to the ferromagnetic transition in martensite at the corresponding Curie temperature, *T_C_^m^*—for *x* = 7, the magnetization increase occurs simultaneously with the MT and shows the temperature hysteresis that is characteristic of this first order transition. It can be also seen in Figure 1 that, for *x* = 6, a second exothermic event is observed upon cooling below the main peak, which corresponds to an intermartensitic transformation (IMT), as will be discussed later.

Table 2 shows, together with the nominal compositions (at %) of the studied alloys, the MT and Curie temperatures (K) for austenite and martensite determined as explained above.

**Figure 3:**
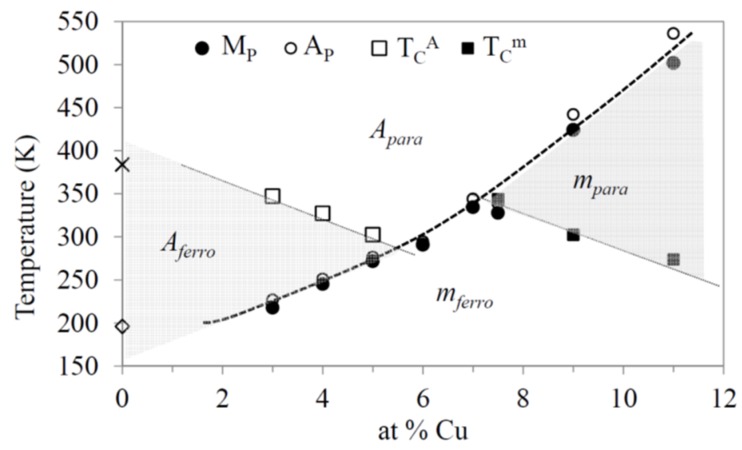
Temperatures of the magnetic and structural transitions as a function of *x*. The regions corresponding to different crystallographic and magnetic states of the alloys are indicated.

Figure 3 shows all the transition temperatures as a function of *x*. The result is a phase diagram where the regions corresponding to different crystallographic and magnetic states of the alloys can be distinguished, similar to that obtained in [18]. Particularly, the domain where the magnetostructural transition *A_para_*⟶*m_ferro_* can occur is restricted to *x* = 5.5–7.5.

### 3.2. Transformation Entropy Changes and Hysteresis

The transformation entropy changes, Δ*S*, were calculated from the DSC curves as follows:

(4) $$\Delta S=\frac{1}{\dot{T}}\int \frac{\dot{Q}}{T}dT.$$

**Figure 4:**
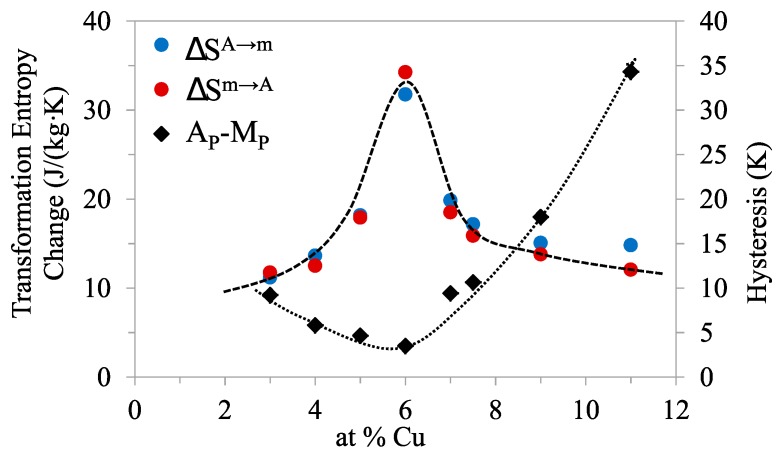
Absolute values of the transformation entropy changes, for the forward and reverse MTs, together with the thermal hysteresis computed as Δ*T* = *A_P_* − *M_P_*, as a function of Cu content.

On the basis of the reproducibility of the obtained values, the accuracy of Δ*S* can be estimated as ±1 J/(kg·K). Figure 4 shows the absolute values of the transformation entropy changes, for the forward and reverse MTs, as a function of composition. It has been previously emphasized that the magnetic contribution adds—in absolute value—to the structural contribution to Δ*S* in cases where the MT produces an increase in magnetization, contrary to what happens for conventional MMSMAs. This is the cause of the increased entropy values, within the window in which the magnetostructural *A_para_*⟶*m_ferro_* transformation occurs. Furthermore, it is widely accepted that |∆*S_mag_*| increases as the temperature gap (*T_C_* − *T_MT_*) does (*T_MT_* is a characteristic MT temperature) [22], reflecting the fact that, the larger this gap, the higher the magnetic order of the ferromagnetic phase. For the studied alloys, the difference between the extrapolated Curie temperature of martensite and the MT temperatures, and thus the magnetic order of the martensite phase when it is formed, is the largest of the set at *x* = 6. Consequently, the magnetic contribution to entropy is also the highest. By the way, the equivalent criteria has been successfully used to analyze the composition dependence of Δ*S* of Co-doped Ni–Mn–Ga alloys; as they undergo *A_ferro_*⟶*m_weak_* MT, Δ *S* decreases with increasing (*T_C_^A^* − *T_MT_*), obviously owing to an increase of the magnetic contribution to the entropy change as the magnetic order of austenite at the MT improves [14].

The temperature hysteresis of the MT, computed as Δ*T* = *A_P_* − *M_P_*, as a function of *x*, is also shown in Figure 4. It can be observed that the hysteresis displays a minimum within the *A_para_*↔*m_ferro_* window, also located at *x* = 6. The fact that the lowest hysteresis coincides with the highest magnetic order in martensite as it forms seems to indicate that magnetism affects the hysteresis. Although this cannot be ruled out [23], other factors that can modify the hysteresis were examined. It was established that minimization of the lattice mismatch between martensite and austenite, which manifests as a close to one value of the middle eigenvalue (λ_2_) of the transformation stretch matrix, leads to low transformation hysteresis [24]. To find if the studied alloys fulfill this hypothesis, the structure of the austenite and martensite phases was determined by means of XRD.

**Figure 5:**
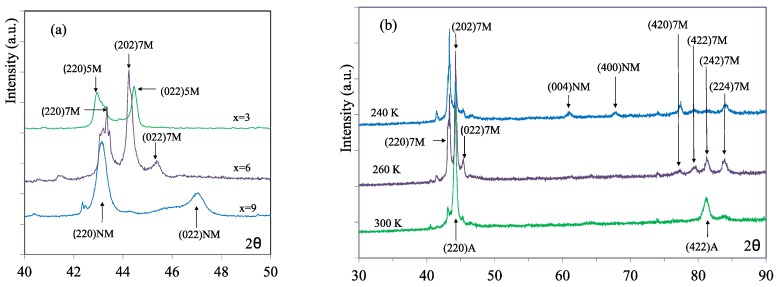
(**a**) Representative portion of the X-ray diffraction (XRD) patterns of the martensite structures for *x* = 3, *x* = 6, and *x* = 9; (**b**) XRD patterns obtained at different temperatures for *x* = 6, corresponding to the structures of austenite, 7M, and NM martensites.

The identification of the Bragg peaks established that, in all cases, the austenite phase was L2_1_ ordered cubic. The martensitic structures are described by body-centered unit cells, not including the modulation, and indexed according to the crystallographic axes of the austenite cubic structure. Depending on the Cu content, different martensite structures were identified. Figure 5a shows a representative portion of the XRD patterns of the martensite structures for *x* = 3, *x* = 6, and *x* = 9. For a low Cu content (*x* = 3, 4), martensite shows a tetragonal five-layer modulated structure, 5M, with *c*/*a* < 1; for a high Cu content (*x* = 7, 9, 11), the structure is identified as non-modulated (NM) tetragonal with *c*/*a* > 1. In the case of *x* = 6, for which the DSC curves revealed a second transformation on cooling, the XRD patterns taken at different temperatures covering the transformation range allow to establish the transformation sequence; the peaks of the L2_1_ structure of the austenite give way on cooling to diffraction peaks corresponding to a first martensite structure that can be identified as orthorhombic, seven-layer modulated, 7M. Additional peaks, corresponding to the tetragonal, non-modulated (NM) martensite, appear at lower temperatures. The XRD patterns obtained at different temperatures for *x* = 6, corresponding to the structures of austenite, and 7M and NM martensites, are shown in Figure 5b. Accordingly, the transformation sequence on cooling is L2_1_→7M→NM. Occurrence of this IMT has been reported in ternary as well as Co- and Cu-doped Ni–Mn–Ga, with the typical feature that the reverse transformation occurs in a single stage [25,26].

**Figure 6:**
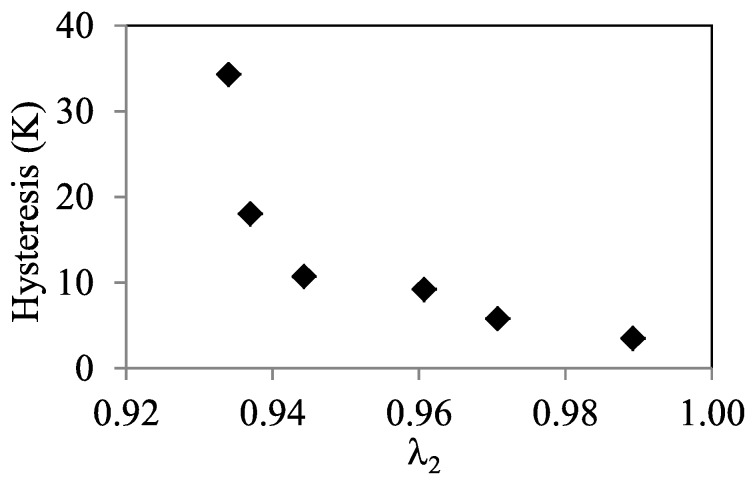
Thermal hysteresis versus middle eigenvalue of the transformation stretch matrix (λ_2_).

The lattice parameters of the identified structures are given in Table 3, together with the tetragonality (*c*/*a*) and the computed middle eigenvalue (λ_2_). The relationship between thermal hysteresis and λ_2_ is shown in Figure 6, where excellent correlation can be observed. Indeed, the lowest λ_2_ is obtained for *x* = 6, pointing to the lattice compatibility as the main cause of the low hysteresis displayed by this alloy. Even so, this issue should be studied more deeply, with additional experiments that allow to analyze in more detail the relationship between crystallographic compatibility and hysteresis and also to assess the role of magnetism.

### 3.3. Magnetostructural Transition Under Applied Field

**Figure 7:**
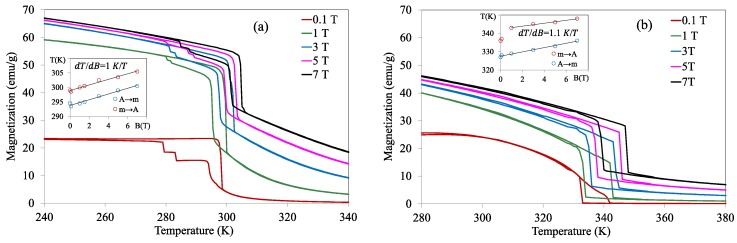
*M*(*T*) curves recorded under different magnetic fields up to 7 T for alloys *x* = 6 (**a**) and *x* = 7 (**b**); the rise of transformation temperatures with the applied field is shown in the insets.

The *M*(*T*) curves recorded under different magnetic fields up to 7 T are shown in Figure 7 for alloys with *x* = 6 (a) and *x* = 7 (b), both within the window in which the magnetostructural *A_para_*→*m_ferro_* transformation occurs. As expected for the positive magnetization jump upon forward MT, the transformation temperatures rise with the increasing applied field, as shown in the insets of Figure 7. The magnetization jumps stabilize above 3 T, at about 28 emu/g for *x* = 6 and 18 emu/g for *x* = 7, and consequently with the decrease in saturation magnetization observed for the increasing Cu content [27]. These data, combined in Equation (1) with the entropy values quoted above, lead to (d*T*/d*H*) rates of 0.8 K/T and 0.9 K/T for *x* = 6 and *x* = 7, respectively, which do not differ much from those obtained experimentally, 1.0 K/T and 1.1 K/T, as shown in the insets of Figure 7.

The temperature derivatives of the *M*(*T*) curves were used to compute the isothermal entropy change associated to MCE, Δ*S_M_*, by means of expressions derived from Maxwell’s relationships, namely,

(5) $$\Delta S_{M}=\int_{0}^{H}\mu_{o}{\left(\frac{\partial M}{\partial T}\right)}_{H}dH.$$

**Figure 8:**
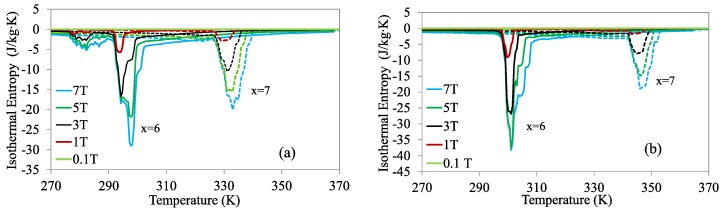
Isothermal entropy change up to different magnetic fields as a function of temperature on cooling (**a**) and heating (**b**) for alloys *x* = 6 (solid lines) and *x* = 7 (dashed lines).

Figure 8a,b display Δ*S_M_* up to different magnetic fields as a function of temperature on cooling and heating, respectively, for both *x* = 6 and *x* = 7 alloys. The results indicate that these alloys display direct and large MCE, with the peak values being greater for the first one. It is worth mentioning that, for the alloy with 6 at% Cu, an additional peak of Δ*S_M_* on cooling reflects the occurrence of the IMT, although only the main peak will be considered.

**Figure 9:**
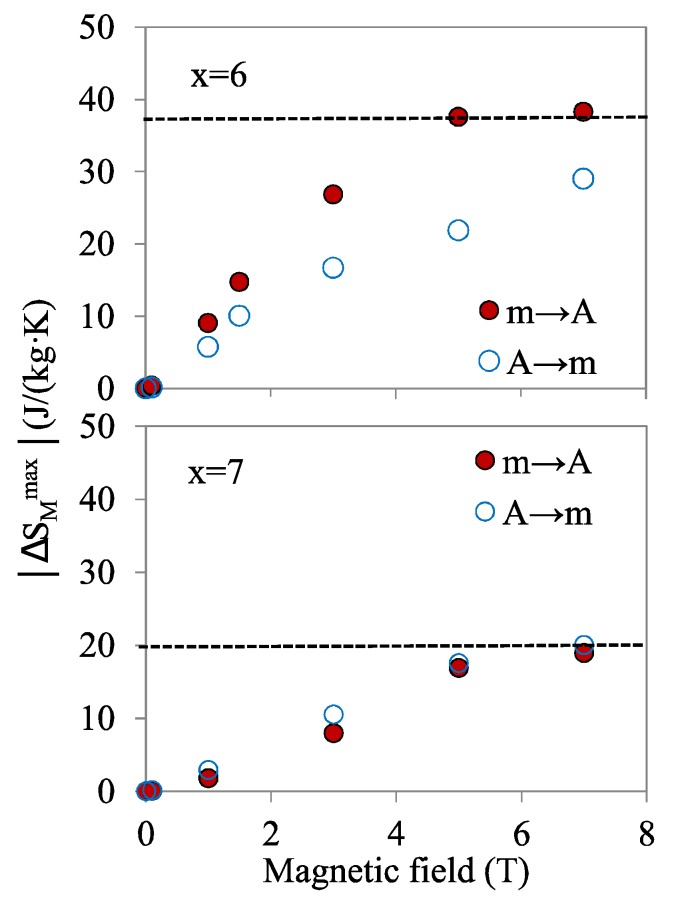
Maximum absolute values of the magnetocaloric entropy change, $\left|\Delta S_{M}^{max}\right|$, as a function of magnetic field, for alloys *x* = 6 and *x* = 7. The dashed lines indicate the corresponding values of the transformation entropy change.

The maximum absolute values of the magnetocaloric entropy change, $\left|\Delta S_{M}^{max}\right|$, for alloys *x* = 6 and *x* = 7 are presented in Figure 9 as a function of magnetic field. For the m→A transition of the *x* = 6 alloy, $\left|\Delta S_{M}^{max}\right|$ saturates for high fields, at a value slightly higher than the zero-field transformation entropy change |∆*S*| ≈ 36 J/(kg·K) (the difference can be attributed to the increase of |∆*S*| with applied field [14]). For the forward transition, A→m, the occurrence of IMT limits the maximum values, although it extends the temperature range for the MCE. In the case of the *x* = 7 alloy, tendency to saturation is also observed, at a value of $\left|\Delta S_{M}^{max}\right|$, close again to the zero-field transformation entropy change for this alloy, |∆*S*| ≈ 20 J/(kg·K).

In this way, the MCE results for these alloys outline that the limit value of the isothermal magnetocaloric entropy is the transformation entropy change |∆*S*| [11]; consequently, the alloy with the largest transformation entropy has the best potential to be used in magnetocaloric effect applications.

### 3.4. Optimization of Caloric Properties

Beyond the maximum value of the entropy and, therefore, the cooling capacity of the material, it is important to consider what value of the applied field is required to produce the maximum temperature change. In the actual cases, the bound value is reached above 5 T, meaning that the material is completely transformed at the peak temperature under that field.

**Figure 10:**
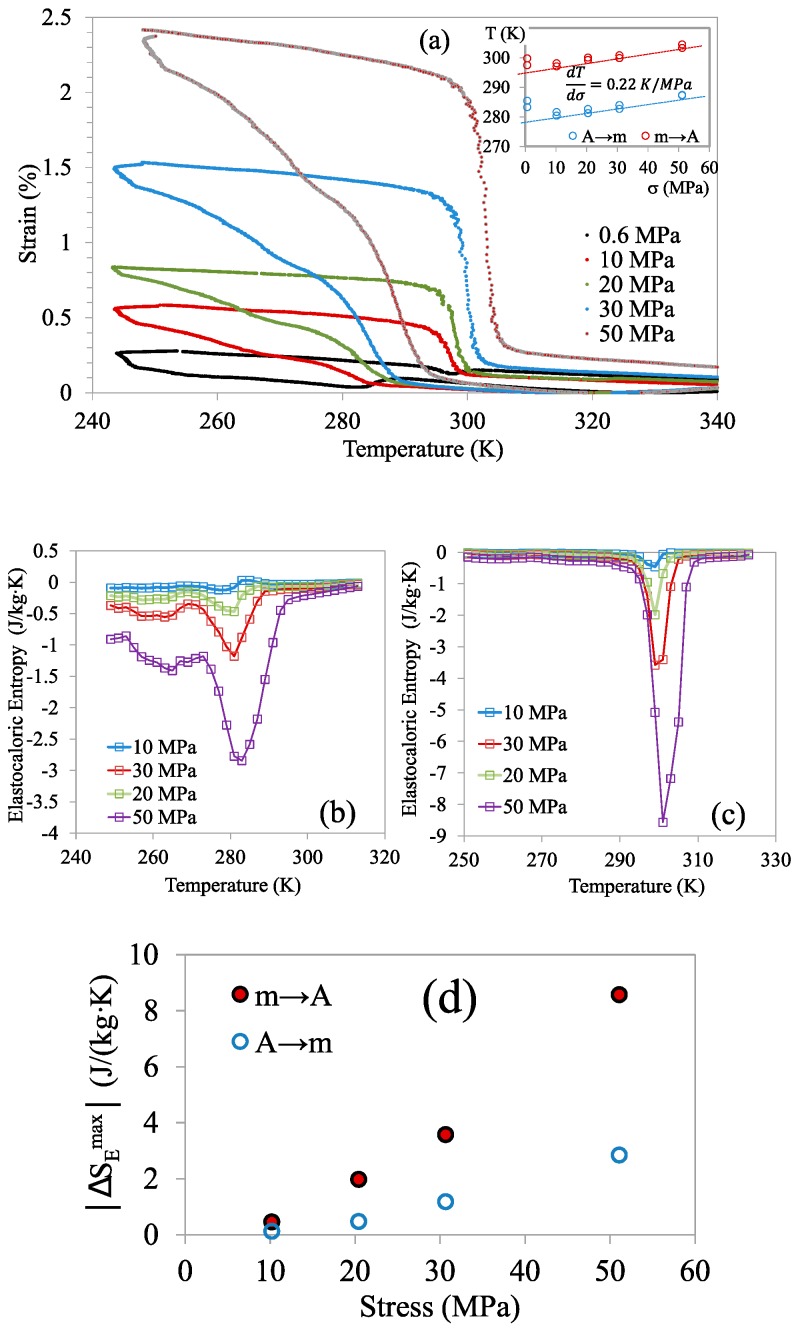
(**a**) ε (*T*) curves under constant loads; the evolution of the transformation temperatures vs. applied stress is shown in the inset. Temperature dependence of the isothermal entropy change for different loads on cooling (**b**) and heating (**c**). (**d**) Maximum absolute values of the elastocaloric entropy change as a function of applied stress.

Indeed, although large values of Δ *S* point to strong caloric effects, according to the Claussius–Clapeyron equation, it also implies that the fields required to induce the MT would be large, confirming that the magnetostructural transition can hardly be induced by the application of a magnetic field like those in commercially available devices; currently, fields of the order of 2 T can be applied using permanent magnets [16]. Because, for alloys undergoing *A_para_*→*m_ferro_* MT, magnetic field and stress act in the same sense, the required magnetic field can be lowered by the application of stress (and vice versa). With this idea in mind, the elastocaloric properties associated with the stress-induced MT were assessed for the alloy with the best prospects, *x* = 6, from the DMA ε(T) curves under constant loads, displayed in Figure 10a. It is worth noting that the IMT on cooling shows up as an additional inflection of strain at low temperatures. The evolution of the transformation temperatures with the applied load is shown in the inset. The isothermal elastocaloric entropy, Δ*S_E_*, is calculated for cooling and heating processes according to

(6) $$\Delta S_{E}=\frac{1}{\rho }\int_{0}^{\sigma }{\left(\frac{\partial \varepsilon }{\partial T}\right)}_{\sigma }d\varepsilon ,$$

where *ε* is the strain, *σ* is the applied stress, and *ρ* is the density of the alloy, which was estimated as 8.32 g/cm^3^ from the XRD results. The temperature dependence of Δ*S_E_* for loads up to 50 MPa is shown in Figure 10b,c. $\left|\Delta S_{E}^{max}\right|$ defined analogously to MCE, is given as a function of applied stress in Figure 10d.

The simultaneous application of magnetic field and mechanical stress would lead to a global shift of the transformation temperatures, given by

(7) $$\Delta T_{MT}=\left(\frac{dT}{dB}\right)\Delta B+\left(\frac{dT}{d\sigma }\right)\Delta \sigma ,$$

where d*T*/d*B* ≈ 1 K/T and d*T*/d*σ*≈ 0.2 K/MPa as given in the insets of Figure 7 and Figure 10a. Of course, this is a mere estimate, because, on the one hand, the values of the slopes determined through the Clausius–Clapeyron relationships correspond to a unique, representative, transformation temperature, and omit the role of dissipation through the martensitic transformation; on the other hand, the effect of the simultaneous application of both magnetic and stress fields may be also affected by magnetostructural coupling and will deserve further study.

As has been emphasized before, ECE and MCE are both direct, and thus can be used simultaneously to improve the overall refrigeration capability of the studied alloy, although the simultaneous use of both caloric effects is not equally advantageous for all working conditions. The values of Δ*S_M_* are among the highest for Heusler-type FSMAs, especially if we focus on materials displaying direct MCE [28,29,30,31,32], and are consistent with reported values for similar compositions in either polycrystalline [20] and single crystalline samples [33]. Regarding the ECE, the obtained values of Δ *S_E_*, significantly lower than those of Δ*S_M_*, are also large compared with those obtained for other Heusler alloys [32,33,34,35]. Large elastocaloric entropy changes under moderate stress values are especially important in Heusler alloys owing to their brittle behavior. Besides, while $\left|\Delta S_{M}^{max}\right|$ saturates above 5 T, meaning that the material is completely transformed, $\left|\Delta S_{E}^{max}\right|$ does not reach saturation for the applied stresses, so that substantially greater loads should be applied to completely transform the material at the working temperatures. An important consequence of the saturation of $\left|\Delta S_{M}^{max}\right|$ is that it limits the enhancement of the global cooling capacity by combination of MCE and ECE, because, if the limit value is reached by application of magnetic field, additional stress will not produce any improvement. However, below saturation, applied stress could promote the advance of the incomplete transformation of the material. For example, $\left|\Delta S_{M}^{max}\right|$ under 3 T is about 60% of the bound value, thus a transformed fraction of 0.6 can be estimated; simultaneous application of 50 MPa would allow transformation of an additional fraction of 0.2.

**Figure 11:**
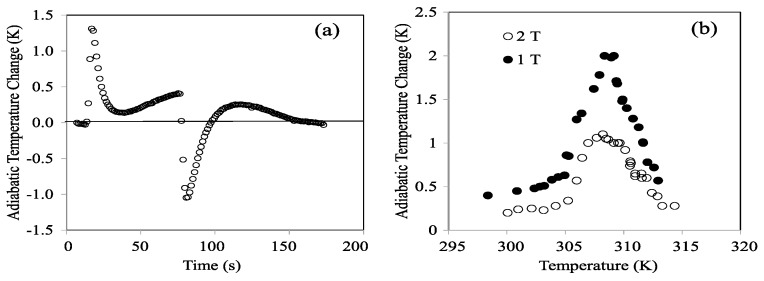
Direct measurements of the adiabatic temperature changes performed (**a**) during compressive loading and unloading at 310 K up to 100 MPa (strain rate of $\dot{\epsilon }=0.01\;s^{-1}$); (**b**) at different temperatures under magnetic field up to 1 and 2 T.

To assess the real potential for caloric cooling, direct measures of adiabatic temperature change (Δ*T_ad_*) were addressed in the *x* = 6 composition, closer to the real conditions used in applications than the indirect calculation of isothermal entropy change through Maxwell’s relationships. Although the measurements are currently in progress, by way of example, some results are shown. Figure 11a shows the adiabatic temperature changes measured during compressive loading and unloading up to 100 MPa at a strain rate $\dot{\epsilon }=0.01\;s^{-1}$. The measurement was performed using a thermocouple attached to a rectangular cuboid-shaped sample at a temperature of *T* = 310 K. On its turn, Figure 11b shows the temperature dependence of the adiabatic temperature change obtained by direct measurements in a magnetic field up to 1 and 2 T. These measurements were made in an own-built set up specially designed for this purpose (see the work of [21] for details).

It’s worth mentioning that, according to Figure 9—and as explained above—application of a 2 T field would promote a maximum transformed fraction of 0.4; similarly, a gross extrapolation to 100 MPa of Figure 10d would yield a maximum entropy between 35% and 40% of the limit value. Therefore, simultaneous application of 2 T and 100 MPa would not exceed 100% transformation and would allow achieving a maximum global cooling of 3.6 K at 310 K.

## 4. Summary and Conclusions

The effect of Mn substitution for Cu in Ni_50_Mn_25−*x*_Ga_25_Cu*_x_* (*x* = 3–11) was analyzed paying attention to the characteristics of the martensitic and magnetic transitions, in order to optimize the functional properties of this alloy system. The main findings of the current study can be summarized as follows:

1. The evolution of the MT temperatures and the Curie temperature as a function of Cu content circumscribes between *x* = 5.5 and *x* = 7.5 the domain of compositions in which the magnetostructural transition *A_para_*↔*m_ferro_* takes place.
2. Within this range of compositions, the measured transformation entropy is increased owing to the magnetic contribution to entropy; contrary to what happens in the case of alloys undergoing *A_ferro_*→*m_weak_* MT, when the MT produces a magnetization increase, the magnetic contribution adds—in absolute value—to the structural contribution. Interestingly, the highest transformation entropy is obtained for *x* = 6, for which the difference between the extrapolated Curie temperature of martensite and the MT temperatures, and thus the magnetic order of the martensite phase as it forms, is the largest of the set.
3. The hysteresis of the MT displays a minimum within the *A_para_*↔*m_ferro_* window, located at *x* = 6. The structural determination made from XRD indicates that the structure of the martensite switches from 5M to NM with increasing *x*, with the singularity that, for *x* = 6, the first formed martensite is the orthorhombic 7M, although it transforms into NM by an IMT on further cooling. The middle eigenvalue of the transformation stretch matrix (λ_2_), computed from the lattice parameters of the identified structures, correlates perfectly with thermal hysteresis, with the closest to one λ_2_ being obtained for *x* = 6. Lattice compatibility is thus considered as the main cause of the low hysteresis displayed by this alloy.
4. The effect of the applied magnetic field on the alloys undergoing *A_para_*↔*m_ferro_* MT is to raise the transformation temperatures, as expected for a positive magnetization jump. However, the d*T*/d*B* rate is low, around 1 K/T, which makes it difficult to induce MT with moderate magnetic fields.
5. The alloys with 6 at% and 7 at% Cu show large and direct MCE, reaching peak values of isothermal magnetocaloric entropy of 36 J/(kg·K) and 20 J/(kg·K), respectively, for fields above 5 T. These bound values coincide with the transformation entropy change of each alloy.
6. To ease the field-induced transformation under low magnetic fields, and also to increase the caloric performance, the effect of mechanical stress was studied for the alloy *x* = 6. A relatively large ECE (compared with other Heusler alloys) with isothermal entropy of 8.5 J/(kg·K) under 50 MPa is observed. Because MCE and ECE are both direct, their combination is expected to improve the overall refrigeration capacity of the alloy.
7. As a concluding remark, the magnetostructural transition for alloy Ni_50_Mn_19_Cu_6_Ga_25_ is accompanied by large transformation entropy change, low thermal hysteresis, and giant MCE and ECE near room temperature, meeting some of the criteria for optimal caloric performance.

## Figures and Tables

**Table 1:** Distinctive characteristics of the magnetostructural transformations *A_ferro_*→*m_weak_* and *A_para_*→*m_ferro_.* MT, martensitic transformation.

Forward MT	∆M	∆S	|∆S|	$\frac{dT}{dH}=-\mu_{o}\frac{∆M}{∆S}$	$\frac{dT}{d\sigma }=-\frac{∆\varepsilon }{∆S}$	$∆S_{M}$	$∆S_{E}$
$A_{ferro}\to m_{weak}$	<0	<0	$\left|\Delta S_{str}\right|-\left|\Delta S_{mag}\right|$	<0	>0	>0	<0
$A_{para}\to m_{ferro}$	>0	<0	$\left|\Delta S_{str}\right|+\left|\Delta S_{mag}\right|$	>0	>0	<0	<0

**Table 2:** Nominal compositions (at %) of the studied alloys, representative temperatures of the MT (*M_P_* and *A_P_*, in K), Curie temperatures for austenite and martensite (*T_C_^A^* and *T_C_^m^*, respectively, in K) together with the absolute values of the transformation entropy changes (|∆*S^A^*^−*m*^| and |∆*S^m^*^−*A*^|, in J·kg^−1^·K^−1^), and temperature hysteresis (*A_P_* − *M_P_*, in K).

Ni	Mn	Ga	Cu	*M_P_*	*A_P_*	*T_C_^m^*	*T_C_^A^*	|∆*S^A^*^−*m*^|	|∆*S ^m^*^−*A*^|	(*A_P_* − *M_P_*)
50	14	25	11	502.0	536.3	273.8	-	16	13	34.3
50	16	25	9	424.2	442.2	302.6	-	16	15	18.0
50	17.5	25	7.5	334.5	343.9	343.7	-	18	17	9.6
50	18	25	7	327.9	337.5	-	-	21	19	9.4
50	19	25	6	291.0	294.5	-	-	35	36	3.5
50	20	25	5	271.8	276.5	-	302.5	19	19	4.7
50	21	25	4	245.3	251.2	-	327.2	14	13	5.8
50	22	25	3	217.7	226.9	-	347.3	12	12	9.2

**Table 3:** Lattice parameters of the identified structures (in nm), tetragonality (*c*/*a*) computed middle eigenvalue (λ_2_), and thermal hysteresis (K) for the different *x* values.

*x*	*a_o_*	*a*	*b*	*c*	*c*/*a*	Martensite	λ_2_	Δ*T*
3	5.825	5.933	-	5.596	0.943	5M	0.961	9.2
4	5.813	5.921	-	5.643	0.953	5M	0.971	5.8
6	5.801	6.042	5.738	5.560	-	7M	0.989	3.5
6	5.801	5.527	-	6.079	1.100	NM	-	-
7	5.804	5.481	-	6.468	1.180	NM	0.944	10.7
9	5.818	5.451	-	6.572	1.206	NM	0.937	18.0
11	5.828	5.443	-	6.576	1.208	NM	0.934	34.3
